# ValuedCare program: a population health model for the delivery of evidence-based care across care continuum for hip fracture patients in Eastern Singapore

**DOI:** 10.1186/s13018-018-0819-9

**Published:** 2018-05-30

**Authors:** Chikul Mittal, Hsien Chieh Daniel Lee, Kiat Sern Goh, Cheng Kiang Adrian Lau, Leeanna Tay, Chuin Siau, Yik Hin Loh, Teck Kheng Edward Goh, Chit Lwin Sandi, Chien Earn Lee

**Affiliations:** 1 Clinical Services, Level 2, Changi General Hospital, 2 Simei Avenue 3, Singapore, 529889 Singapore; 2 ValuedCare Program Office, Changi General Hospital, Singapore, Singapore; 3 Department of Geriatric Medicine, Changi General Hospital, Singapore, Singapore; 4 Department of Orthopaedics, Changi General Hospital, Singapore, Singapore; 5 Executive Office, St. Andrews Community Hospital, Singapore, Singapore; 6 Medical Services, St. Andrews Community Hospital, Singapore, Singapore; 7 Health Services Research, Changi General Hospital, Singapore, Singapore; 8 Executive Office, Changi General Hospital, Singapore, Singapore

**Keywords:** Value-based care, Population health, Hip fracture, Singapore, Evidence-based medicine, ValuedCare, Integrated care, Care pathways

## Abstract

**Background:**

To test a population health program which could, through the application of process redesign, implement multiple evidence-based practices across the continuum of care in a functionally integrated health delivery system and deliver highly reliable and consistent evidence-based surgical care for patients with fragility hip fractures in an acute tertiary general hospital.

**Methods:**

The ValuedCare (VC) program was developed in three distinct phases as an ongoing collaboration between the Geisinger Health System (GHS), USA, and Changi General Hospital (CGH), Singapore, modelled after the GHS ProvenCare® Fragile Hip Fracture Program. Clinical outcome data on consecutive hip fracture patients seen in 12 months pre-intervention were then compared with the post-intervention group. Both pre- and post-intervention groups were followed up across the continuum of care for a period of 12 months.

**Results:**

VC patients showed significant improvement in median time to surgery (97 to 50.5 h), as well as proportion of patients operated within 48 h from hospital admission (48% from 18.8%) as compared to baseline pre-intervention data. These patients also had significant reduction (*p* value < 0.001) of acute inpatient complications such as delirium, pneumonia, urinary tract infections, and pressure sores. VC program has shown significant reduction in median length of stay for acute hospital (13 to 9 days) as well as median combined length of stay for acute and sub-acute rehabilitation hospital (46 to 39 days), thus reducing the total duration of hospitalization and saving total hospital bed days. Operative and inpatient mortality, together with readmission rates, remained low and comparable to international Geriatric Fracture Centers (GFCs).

**Conclusion:**

The implementation of VC methodology has enabled consistent delivery of high-quality, reliable and comprehensive evidence-based care for hip fracture patients at Changi General Hospital. This has also reflected successful change management and interdisciplinary collaboration within the organization through the program. There is potential for testing this methodology as a quality improvement framework replicable to other disease groups in a functionally integrated healthcare system.

## Background

Hip fractures cause significant morbidity and mortality in the elderly. Although the mainstay of treatment is surgical fixation or replacement, these patients are often vulnerable with complex medical, functional, psychosocial issues requiring a multidisciplinary approach to maximize their recovery [1–7].

Singapore is one of the most rapidly aging countries in Asia. The prevalence of adults older than 65 years is set to rise from 9.9% in 2012 to about 20% in the next 20 years [8, 9]. The International Osteoporosis Foundation’s 2009 Asian Audit Report states that the incidence of hip fractures in Singapore is expected to increase from 1300 in 1998 to 9000 per annum by 2050 [9, 10]. Singapore enjoys a unique healthcare ecosystem, where acute episodic care is largely delivered at acute hospitals [AHs (tertiary care corporatized hospitals, fully owned by the government)], while community hospitals (CHs) play an important role in the post-acute rehabilitative care for elderly patients, particularly those with comorbidities. Therefore, the main functions of the community hospitals are to provide geriatric assessment and rehabilitation and ongoing continuation of medical or nursing treatment (sub-acute care) [11]. AH and CH could be independent or vertically integrated co-located organizations with varying degrees of functional or normative integration based on geographical proximities.

AH and CH, together with long-term care facilities and community care resources as well as primary care, aim to work together as a Regional Health System (RHS) to provide seamless integrated care [11–13].

The success of care pathways for hip fractures has been variable [11]. In spite of existing inpatient pathways for patients admitted with hip fractures in Singapore AHs, there are inter- and intra-hospital variations in the level of coordination between different clinical teams (e.g. geriatricians, orthopaedics, anaesthetists, emergency physicians, case managers and therapists), length of stay, time to surgery and bill sizes, often causing fragmentation of care. Additionally, most reported studies take into account only the AH admissions. There is a paucity of data on the effectiveness/long-term outcomes of hip fracture programs across the entire care continuum.

Thus, our aim was to create and test a population health program which could, through the application of process redesign, implement multiple evidence-based medical practices across the continuum of care in an integrated delivery system and deliver highly reliable and consistent evidence-based care for episodic surgical interventions.

Ethical clearance was obtained by the Institutional Review Board (IRB) for this study.

## Methods

### Setting

Changi General Hospital (CGH) is a 1000-bed tertiary acute hospital (AH) located in eastern Singapore. It is part of an Integrated Regional Health System (Eastern Health Alliance) with formal partnerships and a range of healthcare organizations which have a specific focus along the healthcare continuum. St. Andrews Community Hospital (SACH) is one of such partners with a shared purpose of providing seamless integrated care for eastern Singapore [13].

### The ValuedCare methodology

Langley et al. [14] had shown quality improvement through process redesign, and Nolan et al. [15] introduced the concept of high reliability within healthcare. Geisinger’s ProvenCare® [16] had successfully combined both approaches to help address the gap between recommendations and actual clinical practice. In 2014, the ValuedCare (VC) Hip Fracture Program was launched as an ongoing collaboration between the Geisinger Health System (GHS), USA, and Changi General Hospital, Singapore; modelled after the GHS ProvenCare® Fragile Hip Fracture Program [14–21].

The VC Hip Fracture Program was developed in three distinct phases:

- Care gap analysis;
- Process redesign;
- Execution: implementation, capability building, monitoring of compliance and evaluation.

### Care gap analysis

**Fig. 1:**
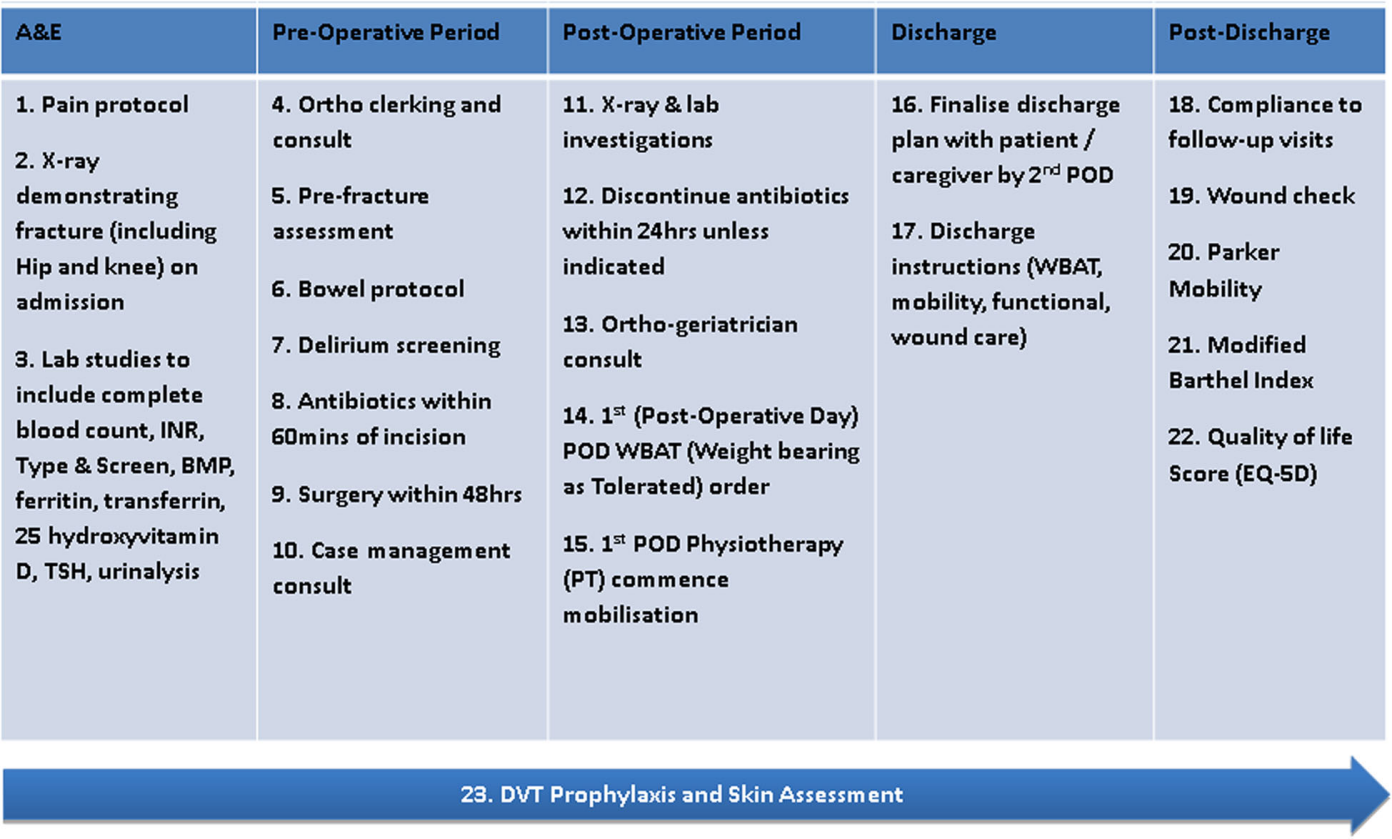
ValuedCare 23 best practices from A&E to inpatient stay and post-discharge care

Facilitated discussions of internationally published evidence and local practice were essential in achieving physician consensus and translating recommendations into local clinical application. To facilitate change management in a multidisciplinary team, clinical champions (senior clinicians and domain experts) were appointed from stakeholder departments including orthopaedics, geriatrics, case management, nursing, allied health and emergency medicine as well as anesthesiology. Clinical champions were actively involved in appraising the evidence together. Level I and II evidence were chosen and discussed, to select 23 best practice elements (BPEs) across the care continuum. Figure 1 summarizes the 23 BPEs; operational definitions and measurable elements were defined for each of the BPEs to ensure compliance.

### Process redesign

Our process redesign began with the fundamental principle of designing and delivering patient-centric care consistently across the entire care continuum. A multidisciplinary team led by the clinical champions worked together to hardwire the evidence-based BPEs into clinical workflows, prevent duplication of services and variability of care, and to manage the health of this patient population, since many of them have multiple co-morbidities. Our intent was to further encourage a productive interaction between informed, activated patients and prepared proactive staff. The team studied current process flows for each discipline and utilized value stream mapping to visualize how multiple disciplines should interact in an integrated pathway.

The finalized care pathway and BPEs were hardwired using information technology and electronic health records (EHR) to enable behavior change and provide decision support, as well as facilitate ease of information diffusion across multiple settings. The aim was to make best care fall within the path of least resistance and in-build a system to minimize unwarranted variations in care.

The care process entailed visualizing the patient journey starting from presentation at the accident and emergency department (A&E), acute hospital admission, community hospital admission and care transition into the community as well as post-discharge specialty care to assess long-term functional outcomes and start secondary prevention.

### Execution

Quality improvement and governance components were deliberately added into the program structure to assure compliance to each of the process elements. All participants knew that compliance with each of the process elements, both as a team and as individuals, would be tracked and real-time feedback given. Due to the strong measurement strategy, any process defect was quickly identified and a focused redesign was immediately started.

For clinical governance, a clinical core team was created to lead the redesign of the care process and implementation of reliable evidence-based care. The multidisciplinary clinical core team monitored compliance to BPEs, information diffusion to ground staff and issues faced on the ground. This team was also responsible for the ownership of clinical outcomes.

The team conducted continuous quality improvement (QI) focus biweekly meetings, where members from different disciplines voice out issues faced on the ground and brainstormed to find solutions and build consensus for immediate remedy or proposed plans for action to address deficiencies in care processes or health outcomes. These meetings worked as mini-PDSA (Plan-Do-Study-Act) cycles and spearheaded the short-term quality improvements within the program. Table 1 summarizes the key improvements achieved as a result of discussions with clinical core team and PDSA cycles.

**Table 1:** Key process improvements achieved through ValuedCare methodology

Objectives	Pre-intervention	Post-intervention
Early surgery within 48 h	Anaesthetic guidelines appended in pathway not used routinely	Orthopaedic team identifies and lists patients for early surgery with the use of anaesthetic checklist upon clerking
	Investigations ordered by the accident and emergency department (A&E) and the orthopaedics team, resulting in missing or duplicate orders	A&E doctor commences investigation order set to facilitate orthopaedics team in review and listing for surgery
	Delayed review of early surgery rates	2 weekly multi-disciplinary review of early surgery rates and documentation of reasons for delayed surgery
	No dedicated high dependency (HD) beds for post-operative care, causing surgical delays	3 dedicated HD beds for ValuedCare patients
Reduce complications	DVT prophylaxis starts from ward admission	DVT prophylaxis starts from A&E
	Ad hoc prescribing of pain, bowel medications, supplements and antibiotics	Standardized electronic orders used by orthopaedics team Medications reviewed by ortho-geriatrician and pharmacist
Restore patient’s functional ability to pre-fracture state	(Post-operative day 1) POD 1 mobilisation by physiotherapist not tightly enforced	POD 1 mobilisation by physiotherapist actively tracked and enforced
	Patient outcome measures acquired only from inpatient stay	Expanded patient outcome measures acquired from both inpatient, outpatient clinic and community hospital over 1 year post-surgery
Enhanced information flow and collection	Manual workflow in documentation with subsequent transcribing to electronic	Electronic documentation in organizational electronic medical record (EMR) system Real-time best practice elements compliance dashboard

The program office provided administrative governance in collaboration with clinical core teams, in order to establish and implement processes to improve care and reduce variation. The program office also facilitated implementation work by addressing barriers and applying quality improvement techniques, which included the development and implementation of appropriate clinical data collection tools, as well as EHR tool build-up and implementation. It was also responsible for planning and implementing the scaling up and spread of the ValuedCare model in AH and CH.

For the strategic support, an oversight/steering committee was created including senior management from both and CH. This platform was useful to review and provide on-going feedback on the progress of the VC program, and also for ‘buy-in’, as senior management from different organizations could come together to share concerns and chart directions. The oversight/steering committee also improved the functional integration between inter-hospital team members.

### Study design

Non-randomized historical controlled study for patients aged 65 years and above treated for single and low-energy hip fractures and undergone hip fracture surgery at AH. A baseline pre-intervention cohort was selected with admission dates between 1 January 2013 to 31 December 2013 (*n* = 351), while the VC cohort comprised of patient admissions between 1 December 2014 to 30 November 2015 (*n* = 329). Patients with multiple fractures, high-impact trauma and pathological fractures (metastasis, avascular necrosis) were excluded from the study.

### Objective

To compare clinical outcomes, complications and healthcare utilization between baseline and ValuedCare groups.

### Statistical analysis

Descriptive epidemiology was used to present demographic variables. Medians were compared using Kruskal-Wallis/Mann-Whitney *U* test. Categorical variables were compared using chi-square test and interval variables using analysis of variance (ANOVA). Statistical calculations were performed using Stata 12.

## Results

The demographic variables and baseline characteristics between pre-intervention and VC patients are reported in Table 2. The two groups had comparable representations in age, gender, race, pre-fracture residence and pre-fracture mobility.

**Table 2:** Demographics and pre-morbidity profile baseline and ValuedCare groups

Variable	Baseline (*n* = 351)	ValuedCare (*n* = 329)	*p* value
Age			
Mean (SD)	81.11 (8.0)	80.35 (7.4)	0.20
Median (min–max)	82 (65–99)	81 (65–102)	0.85
Age group, *n* (%)			0.013
65–74	91 (25.9%)	78 (23.7%)	
75–84	130 (37.0%)	157 (47.7%)	
≥ 85	130 (37.0%)	94 (28.6%)	
Gender, *n* (%)			0.617
Females	247 (70.4%)	225 (68.4%)	
Males	104 (29.6%)	104 (31.6%)	
Race, *n* (%)			0.557
Chinese	265 (75.5%)	247 (75.1%)	
Malays	49 (14.0%)	51 (15.5%)	
Indians	20 (5.7%)	12 (3.6%)	
Others	17 (4.8%)	19 (5.8%)	
Pre-fracture residence, *n* (%)			0.77
Home	332 (94.6%)	312 (94.8%)	
Nursing home	14 (4%)	12 (3.6%)	
Sheltered home	5 (1.4%)	5 (1.5%)	
Pre-fracture aid, *n* (%)			0.152
No aid	187 (53.3%)	197 (61%)	
Walking aid	157 (44.7%)	118 (36.5%)	
Wheelchair	7 (2%)	6 (1.9%)	
Bedbound	0	1 (0.3%)	
Others	0	1 (0.3%)	
Hypertension	188 (53.6%)	228 (69.3%)	< 0.001
Hyperlipidaemia	99 (28.2%)	145 (44.1%)	< 0.001
Diabetes mellitus	130 (37.0%)	111 (33.7%)	0.379
Ischaemic heart disease (IHD)	68 (19.4%)	51 (15.5%)	0.191
CCF/heart failure	11 (3.1%)	12 (3.6%)	0.83
COPD/cold/asthma	15 (4.3%)	17 (5.2%)	0.593
Peripheral vascular disease (PVD)	13 (3.7%)	5 (1.5%)	0.095
Chronic renal failure	50 (14.2%)	23 (7.0%)	0.03

Table 3 compares outcomes between baseline pre-intervention and VC populations. The inpatient, 30-day post-discharge and 12-month post-discharge mortality remained low at 1.2, 1.2 and 8% respectively; mortality data was obtained from a national registry. Forty-eight percent of the VC patients received surgical treatment within 48 h from admission, compared to 18.8% at baseline. Median time to surgery was reduced from 97 to 50.5 h. Early access to surgery has shown to improve clinical outcomes in several international studies [5, 10]. VC patients also had less acute inpatient complications as compared to the baseline pre-intervention cohort, in particular lower rates of delirium, pneumonia, urinary tract infections (UTI) and pressure sores. A reduction in 30-day and 180-day readmission rates for hip fracture-related causes was noticed, although not statistically significant.

**Table 3:** Comparison of clinical outcomes between baseline and ValuedCare patients

Mortality rates			
Variable	Baseline (*n* = 351)	ValuedCare (*n* = 329)	*p* value
Index inpatient mortality, *n* (%)	6 (1.7%)	4 (1.2%)	0.75
Variable	Baseline (*n* = 344*)	ValuedCare (*n* = 325*)	*p* value
Post-discharge 30 days mortality rate, *n* (%)	1 (0.3%)	4 (1.2%)	0.2
Post-discharge 12 months mortality rate, *n* (%)	27 (7.8%)	26 (8.0%)	0.94
Acute hospital inpatient complications			
Variable	Baseline (*n* = 351)	ValuedCare (*n* = 329)	*p* value
Wound infection	1 (0.3%)	0 (0.0%)	0.5136
Implant failure	4 (1.1%)	1 (0.3%)	0.374
Delirium	36 (10.3%)	13 (4.0%)	0.002
Acute retention of urine (ARU)	53 (15.1%)	34 (10.3%)	0.067
Pneumonia	45 (12.8%)	13 (4.0%)	< 0.001
Urinary tract infection (UTI)	90 (25.6%)	16 (4.9%)	< 0.001
Pressure sore	39 (11.1%)	1 (0.3%)	< 0.001
Deep vein thrombosis	4 (1.1%)	8 (2.4%)	0.250
Pulmonary embolism	5 (1.4%)	2 (0.6%)	0.452
Acute myocardial infarction	8 (2.3%)	5 (1.5%)	0.580
Stroke	4 (1.1%)	2 (0.6%)	0.687
Readmission rates	Baseline (*n* = 344*)	ValuedCare (*n* = 325*)	
30-day readmission (all cause), *n* (%)	26 (7.6%)	31 (9.5%)	0.36
30-day readmission (hip fracture related), *n* (%)	17 (4.9%)	12 (3.7%)	0.42
180-day readmission (all cause), *n* (%)	74(21.5%)	72 (22.2%)	0.84
180-day readmission (hip fracture related), *n* (%)	44 (12.8%)	27 (8.3%)	0.06
Time to surgery	Baseline (*n* = 351)	ValuedCare (*n* = 329)	
Surgery within 48 h from time of decision to admit	66 (18.8%)	158 (48.0%)	< 0.001
Time to surgery, h			< 0.001
Mean (SD)	119.2 (86.3)	70.63 (64.4)	
Median (min–max)	97 (11–499)	50.5 (0.11–638)	

*Inpatient deaths and discharge against advice cases are taken out of the analysis

**Fig. 2:**
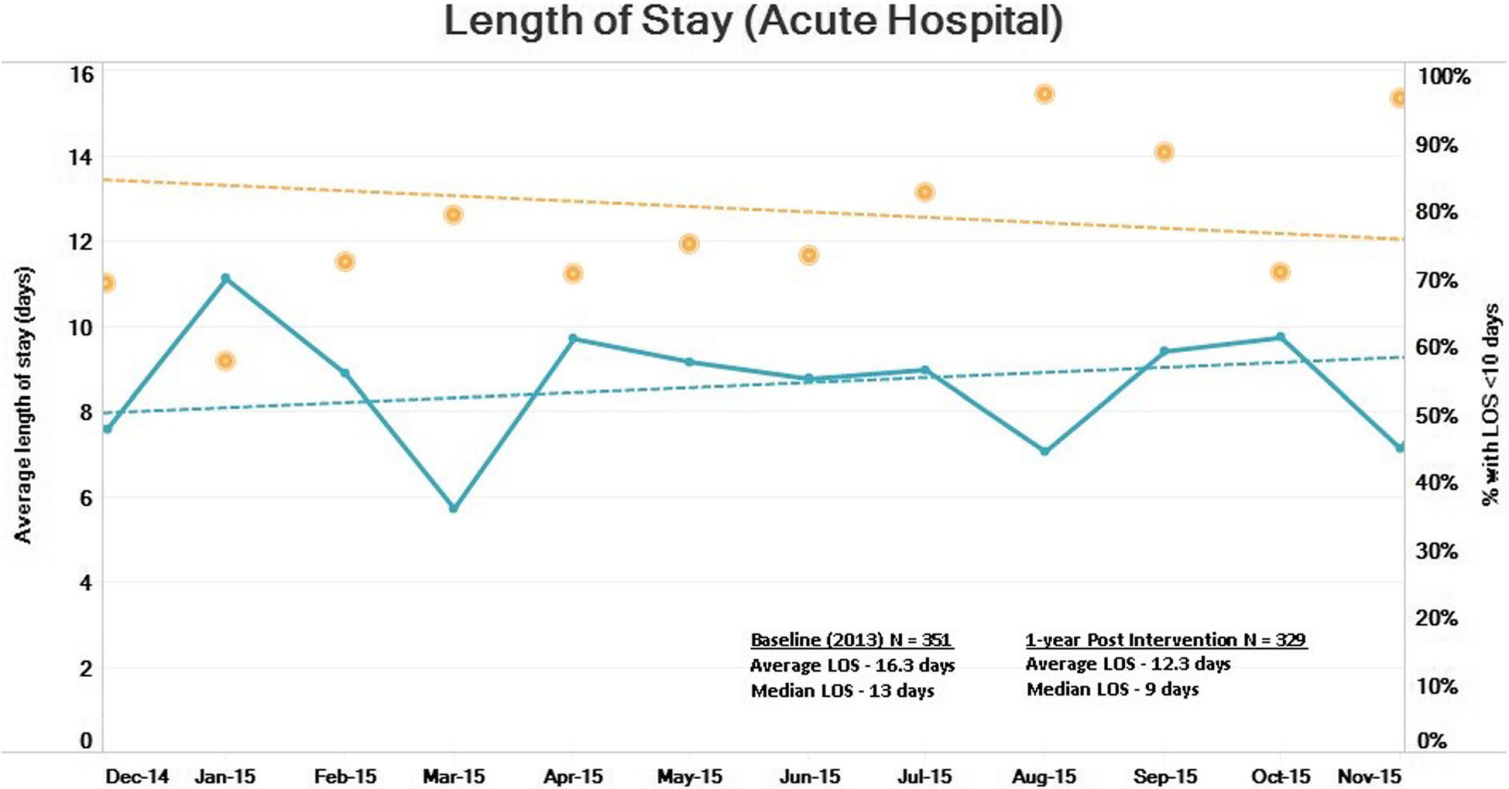
Length of stay (acute hospital)

**Fig. 3:**
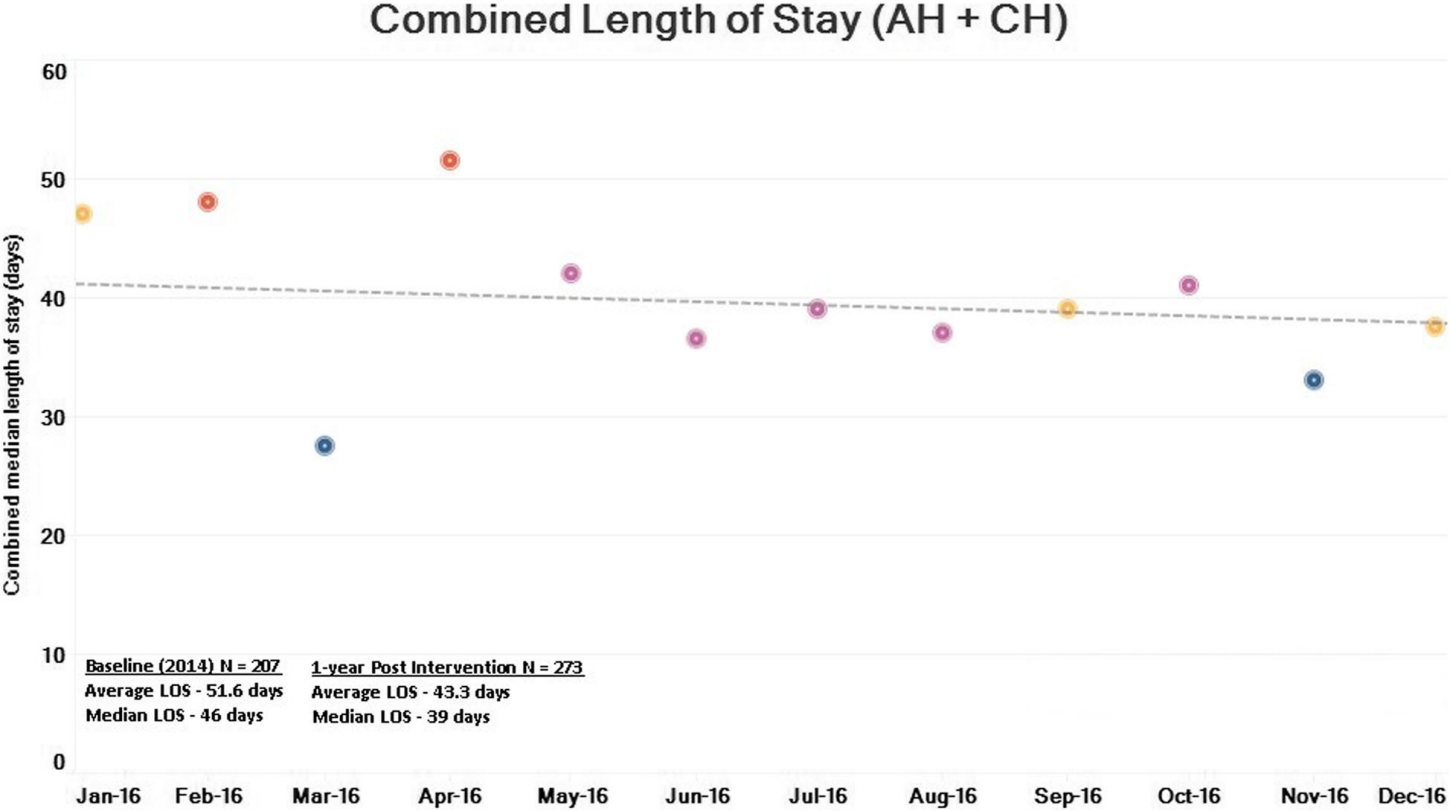
Combined length of stay (AH + CH). Combined average LOS distribution in days

Comparisons of length of stay (LOS) between pre- and post-intervention data at AH and AH-CH combined are summarized, respectively, in Figs. 2 and 3. A LOS of ≤ 10 days for AH was taken as an internal reference target at the beginning of the program after a review of relevant literature [3, 22]. For AH (Fig. 2), the reduction in LOS was noticed to be statistically significant at a *p* value < 0.001. Figure 2 also shows a decreasing trend in AH monthly average LOS for a 1-year post-intervention period (*p* value < 0.001, *R*^2^ = 0.452) and a corresponding increasing trend for a percentage of patients with LOS ≤ 10 days (*p* value < 0.001, *R*^2^ = 0.522).

Implementation of VC in CH began 1 year later than that in AH; thus, pre- and post-intervention data for combined LOS were studied a year later as compared to AH data (Fig. 3). There was a significant reduction in combined LOS (AH + CH) between baseline and post-intervention population values (*p* value < 0.001). A decreasing trend in monthly average LOS for a 1-year post-intervention period (*p* value < 0.001, *R*^2^ = 0.32) was also noticed.

## Discussion

As Michael Porter describes, value cannot be understood at the level of a hospital, a care site, a specialty, or an intervention. Value is created in caring for a patient’s medical condition over the full cycle of care [21]. VC program attempts to implement system level transformation to measure health outcomes over disease life cycle, including functional outcomes, and cost of care over care continuum across institutions. The focus thus shifts to providing evidence-based and consistent patient care to achieve better outcomes over the entire cycle of care, instead of merely introducing cost containment measures within a particular institution. VC program aims to totally study the overall healthcare ecosystem (rather just hospital-based services), so that true efficiencies are achieved to create value for patients (e.g. right siting of services and elimination of non-value adding services). VC BPEs include preventive interventions such as osteoporosis treatment, secondary fall prevention, functional rehabilitation and caregiver training. It also measures longer term functional health outcomes such as longitudinal Modified Barthel’s Index (MBI)/Barthel and Functional Independence.

We compared the results of our VC program with the published results of international Geriatric Fracture Centers (GFCs) in terms of LOS, mortality and morbidity data (Table 4). Among the models compared, ValuedCare’s mortality rates of 1.2 and 8% were the lowest for, respectively, intra-hospital rates and at 1 year follow-up. One-year mortality for surgically managed hip fracture cases has been reported in some overseas centres to be as high as 30% [23]. Our 30-day readmission rates are also among the lowest. It was noticed in the international literature that LOS can vary between 4.2 and 15.8 days. Several factors affect LOS, such as differences in models of step-down care available in different countries, efficiency of referral mechanisms, level of integration, as well as availability of step-down or rehabilitation care beds. In Rochester [24], where the LOS is the shortest (4.2 days), patients are moved to their nursing facility rehabilitation beds by the third post-operative days. In most centres, including Singapore, patients have to wait for an available bed in rehabilitation hospital or CH. Under the VC program, AH and CH teams worked together to streamline the inter-hospital transfer process and eliminate unnecessary administrative steps. Referral requests were raised early (post-operative day 2), and specific referral and acceptance criteria were delineated and made transparent to the clinical team. CH rehabilitation team reviewed the patient at AH and hastened the transfer process if patients met the predefined criteria.

**Table 4:** Comparison of length of stay, morbidity and mortality data of various geriatric hip fracture programs [8, 23–26]

	Rochester model [24]	Innsbruck model [27]	Singapore ValuedCare	Hong Kong model [28]	National Hip Fracture Database (UK) [8, 22]
Length of stay, days	4.2	11.3	9	6.4	15.8
30-day readmission	9.8	5.2	3.7	15	11.8
Hospital mortality rates, %	1.6	3.1	1.2	1.25	8.2
1-year mortality, %	21.2	Not available	8	16.4–22.4	19.3

It was noticed that after VC implementation, the average length of stay (ALOS) at AH, as well as combined LOS (AH + CH), has shown consistent significant improvement. ALOS at AH decreased from 16.3 to 12.3 days (median 13 to 9 days), while combined ALOS (AH + CH) showed a reduction from 51.6 to 43.3 days (median 46 to 39 days). Reduction in combined length of stay is of relevant importance as it indicates that this result was achieved with process redesign instead of shifting the burden of care to the next healthcare setting. Reductions in LOS, along with reduction in hip fracture-related admissions up to 6 months (12.8 to 8.3%), result in savings in hospital bed days. This is significant especially in the context of rising demand for hospital beds and costs with a growing ageing population.

In both AH and CH, rehabilitation doctors, nurses, therapists and medical social workers work together to speed up the recovery process. Early active walking exercise and post-discharge rehabilitation by community nurses and therapists play an important role in shortening the need for inpatient treatment. The regular assessments of the mental and functional state can help to enhance the recovery of patients. The medical social workers identify social or financial problems that may complicate the discharge and activate available resources to help them. The overall shortened hospital stay reflects the effectiveness and cooperation with this multidisciplinary approach.

VC results are consistent with our hypothesis that with implementation of BPEs and minimizing unwarranted variations in care, health and utilization outcomes can be improved. The team acknowledges the scope for further improvements in the combined LOS in AH + CH. We are working towards achieving our target of getting at least 70% VC cases operated within 48 h from hospital admission. Also, detailed analysis of functional outcomes, quality of life (QOL) and interventions for secondary prevention of falls and fractures will be performed within the next phase of VC program.

## Conclusion

The implementation of VC hip fracture program has enabled consistent delivery of high-quality, reliable and comprehensive evidence-based care for hip fracture patients. This has been further strengthened by successful change management and interdisciplinary collaboration within the organization. Efforts are on-going to sustain these best practices and augment gains. The program will be scaled to include other medical conditions using VC methodology which could, through the application of process redesign, implement multiple evidence-based medical practices across the continuum of care in an integrated delivery system and deliver high-quality reliable care.

### Limitations

Non-randomized historical controlled study design has inherent limitations, but a randomized control trial presents ethical concerns as the ‘intervention’ aim consists of implementing best practice elements. The implementation of VC program in CH was a year later as compared to that in AH; thus, pre- and post-intervention data for CH LOS was studied a year later as compared to AH data. Readmission data were specific to AH as we do not have data for readmissions to other hospitals.

## Abbreviations

AH: Acute hospital
ALOS: Average length of stay
CGH: Changi General Hospital
CH: Community hospital
EHA: Eastern Health Alliance
EHR: Electronic health records
MBI: Modified Barthel’s Index
MOH: Ministry of Health Singapore
QI: Quality improvement
RHS: Regional Health System
SACH: St. Andrews Community Hospital
VC: ValuedCare
